# Supplementary material for: The Systems Biology Research Tool: evolvable open-source software
Source: BMC Syst Biol. 2008 Jun 29;2:55. doi: 10.1186/1752-0509-2-55 (PMC2446383; doi:10.1186/1752-0509-2-55)
Supplement: Additional file 1 — SBRT Archive. An archive of the current version of the Systems Biology Research Tool. [file 1752-0509-2-55-S1.zip › sbrt-1.4.0/doc/developers_guide/api/sbrt/kernel/util/package-tree.html]

sbrt.kernel.util Class Hierarchy


|  |  |  |
| --- | --- | --- |
| **Overview** \| **Package** \| Class \| Use \| **Tree** \| **Deprecated** \| **Index** \| **Help** | |  |
| **PREV**   **NEXT** | **FRAMES**    **NO FRAMES**     **All Classes** |  |


---


## Hierarchy For Package sbrt.kernel.util

**Package Hierarchies:**: All Packages

---

## Class Hierarchy

- java.lang.Object
  - sbrt.kernel.util.**AbstractProgressMonitor** (implements sbrt.kernel.util.MutableProgressMonitor)
    - sbrt.kernel.util.**DiscreteProgressMonitor**- sbrt.kernel.util.**PercentProgressMonitor**
    - sbrt.kernel.util.**BooleanRelation**<E> (implements java.lang.Iterable<T>)
      - sbrt.kernel.util.**And**<E>- sbrt.kernel.util.**Or**<E>
      - sbrt.kernel.util.**UnmodifiableCollection**<E> (implements java.util.Collection<E>)- sbrt.kernel.util.**UnmodifiableIterator**<E> (implements java.util.Iterator<E>)- sbrt.kernel.util.**UnmodifiableList**<E> (implements java.util.List<E>)- sbrt.kernel.util.**UnmodifiableMap**<K,V> (implements java.util.Map<K,V>)- sbrt.kernel.util.**UnmodifiableSet**<E> (implements java.util.Set<E>)- sbrt.kernel.util.**Util**

## Interface Hierarchy

- sbrt.kernel.util.**ProgressMonitor**
  - sbrt.kernel.util.**MutableProgressMonitor**

---


|  |  |  |
| --- | --- | --- |
| **Overview** \| **Package** \| Class \| Use \| **Tree** \| **Deprecated** \| **Index** \| **Help** | |  |
| **PREV**   **NEXT** | **FRAMES**    **NO FRAMES**     **All Classes** |  |


---
